# HDAC Inhibitors Repress BARD1 Isoform Expression in Acute Myeloid Leukemia Cells via Activation of miR-19a and/or b

**DOI:** 10.1371/journal.pone.0083018

**Published:** 2013-12-11

**Authors:** Ilaria Lepore, Carmela Dell’Aversana, Maxim Pilyugin, Mariarosaria Conte, Angela Nebbioso, Floriana De Bellis, Francesco P. Tambaro, Tiziana Izzo, Guillermo Garcia-Manero, Felicetto Ferrara, Irmgard Irminger-Finger, Lucia Altucci

**Affiliations:** 1 Department of Biochemistry, Biophysics and General Pathology, Seconda Università degli Studi di Napoli, Naples, Italy; 2 Institute of Genetics and Biophysics (IGB), Naples, Italy; 3 Department of Leukemia, The University of Texas MD Anderson Cancer Center, Houston, Texas, United States of America; 4 Molecular Gynecology and Obstetrics Laboratory, Department of Gynecology and Obstetrics and Department of Genetic and Laboratory Medicine, University Hospitals of Geneva, HUG, Genève, Switzerland; 5 Division of Hematology and Stem Cell Transplantation Unit, Cardarelli Hospital, Naples, Italy; Université Paris-Diderot, France

## Abstract

Over the past years BARD1 (BRCA1-associated RING domain 1) has been considered as both a BRCA1 (BReast Cancer susceptibility gene 1, early onset) interactor and tumor suppressor gene mutated in breast and ovarian cancers. Despite its role as a stable heterodimer with BRCA1, increasing evidence indicates that BARD1 also has BRCA1-independent oncogenic functions. Here, we investigate BARD1 expression and function in human acute myeloid leukemias and its modulation by epigenetic mechanism(s) and microRNAs. We show that the HDACi (histone deacetylase inhibitor) Vorinostat reduces BARD1 mRNA levels by increasing miR-19a and miR-19b expression levels. Moreover, we identify a specific BARD1 isoform, which might act as tumor diagnostic and prognostic markers.

## Introduction

One of the most important goals of current anti-cancer research is to identify new diagnostic markers, allowing both early screening of patients and their potential stratification for personalized medicine. The elucidation of pathways frequently altered in cancer, such as proliferation, apoptosis, DNA repair and differentiation, is thus of vital importance [1]. Over the past years increasing evidence has emphasized the involvement of BARD1 (BRCA1-associated RING domain 1) in the pathogenesis of different cancers, including breast, ovarian, uterine, colon and lung cancer, as well as neuroblastoma [2-6]. BARD1, well known as a BRCA1 partner, is considered a tumor suppressor due to its impact on cell cycle progression, apoptosis, gene expression regulation, RNA processing and DNA repair mechanisms, either conjugated with BRCA1 in a stable heterodimer through the interaction of their respective RING finger domains, or in its monomeric form [7-16].

Therefore, the presence of point mutations in BARD1, or deletion-bearing or truncated BARD1 isoforms, generated by alternative splicing of mature mRNA transcripts or by the use of multiple translation start sites (TSS), respectively, might significantly affect its functions, leading to cancer development. However, overexpressed aberrant isoforms rather than mutations of BARD1 have been reported in cancer. *Li* et al described the characterization of different BARD1 isoforms in breast, ovarian and uterine carcinomas, which are absent or strongly down-regulated in healthy tissues, suggesting that these isoforms might be a predisposing factor or may result from the development of the disease [3,17].

Interestingly, and consistent with its function as a tumor suppressor, the loss of FL BARD1 (Full Length BARD1) in favor of mutated variants is a commonly reported event in solid cancers. For instance, high levels of FL BARD1 correlate with a good prognosis in colon cancer patients, whereas BARD1 splicing isoforms are variably expressed both in normal and cancer cells [4], and the expression of epitopes, consistent with isoform but not FL BARD1 expression, was correlated with poor survival [5]. BARD1 isoforms have also been detected in NSCLC (Non-Small Cell Lung Cancer) samples and their expression correlated with decreased survival [5].

BARD1 expression has been found to be down-regulated in samples from myelodysplastic syndrome patients (MDS) with progression to acute myeloid leukemia (AML) [18]. This prompted us to investigate the role of BARD1 isoforms in AML and to analyze its possible pharmacological modulation for medical intervention.

In the last few years, several epigenetic modulating compounds have been designed and applied to cancer treatment or used in preclinical development. To date, the most common epigenetic compounds are histone deacetylase (HDAC) inhibitors, the so-called HDACi. Approved in 2006 by the FDA for the second line treatment of cutaneous T-cell lymphoma (CTCL), *suberoylanilidehydroxamic acid* (SAHA, *Vorinostat*), is the best known class I & II HDACi [19-22]. Additional class-specific modulators include the class I-specific HDACi Entinostat (MS-275) [20,23-26], the class II inhibitor MC 1568 [27,28] and the HDAC6 inhibitor ST-80 [29].

Other key actors involved in gene expression control are microRNAs (miRNAs), small non-coding RNAs able to bind specific mRNA target sequences. miRNAs recognize a complementary sequence within the 3’ untranslated region (3’UTR) of a specific mRNA, inducing degradation of the target when a full match miRNA-mRNA occurs, or blocking translation in the event of an incomplete complementation [30-35].

The correlation between miRNAs and epigenetic enzyme action is complex given that miRNA gene promoters may contain CpG islands, the targets of epigenetic control of gene expression by DNMTs (DNA-methyl transferases) and HDACs [36,37].

We investigated BARD1 expression and function in AML and its modulation by epigenetic mechanisms and miRNA pathways. We found that Vorinostat reduces BARD1 mRNA levels by increasing miR-19a and miR-19b expression. Moreover, we identified specific BARD1 isoforms that might act as oncogenes and as tumor diagnostic and prognostic markers.

## Materials and Methods

### Chemical compounds

Vorinostat (SAHA, Merck), Entinostat (MS-275, Alexis), MC 1568 and ST 80 were dissolved in DMSO (Di-Methyl SulfOxide, Sigma-Aldrich) and used at a final concentration of 5 μM.

### AML *ex vivo* samples

AML blasts were recovered bone marrow and purified by Ficoll (Sigma-Aldrich) density gradient separation: after a centrifugation of 25 minutes at 1250 rpm, the layer of mono-nuclear cells was diluted in cell culture medium and further centrifuged for 5 minutes at the same speed. Cell pellet was dissolved in RPMI 1640 (EuroClone) enriched with 20% heat-inactivated FBS (Fetal Bovine Serum, Sigma Aldrich), 1% glutamine, 1% penicillin/streptomycin and 0.1% gentamycin (EuroClone), and kept at 37°C in air and 5% CO_2_. The ethical committee of the Seconda Università degli Studi di Napoli approved these experiments carried out after written informed consent from the donors.

### Cell lines

Human leukemia cell lines (U937, NB4, K562 and HL60) were grown in RPMI 1640 medium (EuroClone) supplemented with 10% heat-inactivated FBS (Sigma Aldrich), 1% glutamine, 1% penicillin/streptomycin and 0.1% gentamycin (EuroClone), and kept at 37°C in air and 5% CO_2_. Conversely, adherent HeLa (human epithelial cervical cancer), MCF7 (human breast cancer) and Kelly (human neuroblastoma) cells were grown in D-MEM medium (EuroClone) supplemented with the components described above and in the same settings.

### RNA extraction

Cells were collected by centrifugation and resuspended in 1 ml of TRIzol reagent (Invitrogen), strongly shaken and stored at -20°C overnight. The following day the samples were supplemented with 100 µl of 2-bromo-3-chloro propane (Sigma Aldrich), gently shaken and incubated for 15 minutes at RT. After a centrifugation of 15 minutes at 12000 rpm at 4°C the supernatants were put in a fresh tube and supplemented with 500 µl of cold isopropylicalcohol. RNA precipitation reaction was carried out for 30 minutes at -80°C and followed by a centrifugation of 30 minutes at 12000 rpm at 4°C. The pellets were then resuspended in 1 ml of cold 70% ethanol and the samples were centrifuged again for 10 minutes at 7500 rpm at 4°C. The pellets were subsequently dried at 42°C for a few minutes and resuspended in DEPC-treated H_2_O. RNA samples were quantified with a Nanodrop 1000 spectrophotometer and their quality was evaluated using Agilent RNA 6000 Nano Assay.

### RT-PCR

RNA samples were converted into cDNA using VILO Invitrogen kit: 1 µg of RNA was mixed with 1x VILO Reaction Mix, 1x Super-Script Mix and DEPC-H_2_O; the samples were then incubated for 10 minutes at 25°C, 60 minutes at 42°C and 5 minutes at 85°C. RT-PCR experiments for BARD1 exon 6 – exon 11 and GAPDH were then performed with 75 ng of cDNA in presence of a reaction mixture containing: 2.5 mM MgCl_2_, 0.4 mM dNTP, 0.5 µM of each primer, 1.5 U of Taq GOLD DNA polymerase, 1x GOLD buffer (Applied Biosystem), sterile H_2_O. The thermal protocol was as follows: 95°C for 10 minutes plus 30 cycles at 95°C for 30 seconds, 60°C for 30 seconds and 72°C for 45 seconds, with a final elongation of 10 minutes at 72°C. BARD1 exon 1 – exon 11 amplification was performed with a different thermal protocol: 95°C for 10 minutes plus 35 cycles at 95°C for 1 minute, 56°C for 1 minute and 72°C for 2 minutes with a final elongation of 10 minutes at 72°C. Primers used: forward exon 1 5’ GAGGAGCCTTTCATCCGAAG 3’ (-28 from ATG); forward exon 6 5’ CTCCAGCATAAGGCATTGGT 3’ (+1441 from ATG); reverse exon 11 5’ CGAACCCTCTCTGGGTGATA 3’ (+2252 from ATG). GAPDH quantification was used as normalization control: forward primer 5’ GGAGTCAACGGATTTGGTCGT 3’, reverse primer 5’ GCTTCCCGTTCTCAGCCT TGA 3’. 

### 5’ RACE PCR

5’ RACE PCR of BARD1 isoforms was performed using 5’ RACE System for Rapid Amplification of cDNA Ends Kit (Invitrogen) according to manufacturer’s instructions. The experiments were carried out with 3 µg of NB4 RNA samples. RACE primers in exon 6: GSP1 5’ GGCTCCATAGGAAAGTAA 3’ (+1554 from ATG), GSP2 5’GACTATATCCACATGCCCA TTC 3’ (+1530 from ATG), GSP3 5’ CATCGTGAAGTGGTGAGTCATT 3’ (+1501 from ATG). Subsequently 5' RACE products were run on 1% agarose gel, recovered with a QIAquick Gel Extraction Kit (Qiagen) and used to perform the TOPO TA Cloning® reaction (Invitrogen): 4 µl of PCR product were mixed with 10 ng of the pCR®II-TOPO® vector (Invitrogen) in presence of a salt solution (200 mM NaCl, 10 mM MgCl_2_), and the reaction was carried out for 1 hour at RT. The plasmids obtained were amplified in JM109 super competent *E. coli* (Invitrogen) and were extracted with MINIprepNucleoSpinPlasmid (Macherey-Nagel). 1 µg of plasmid was then digested with 10 U of EcoRI enzyme (Biolabs) for 1 hour and 30 minutes to ensure the presence of the PCR product in the vector. Finally, DNA samples were sent to the sequencing service, which utilized M13 forward and reverse primers to obtain the sequences. 

### miRNA target prediction analysis

A computational analysis of the predicted miRNAs to target BARD1 3’UTR region was performed within the miRBase database and a list of all miRNAs that may target BARD1 was obtained by selecting miRNAs commonly indicated by ‘Target Scan’, ‘Miranda’ and ‘PictarVert’ tools.

### miRNA microarray

Human miRNA microarray experiments were performed using LC Sciences Service (LC Sciences, Austin, USA), based on µParaflo^®^Microfluidic Biochip Technology, containing 723 mature human miRNA probes (Sanger miRBase, release 10.1). NB4 cells were stimulated for 6 hours with 5 µM Vorinostat. After RNA extraction, 1 µg of untreated (NT) and treated (s) samples was labeled with Cy3 or Cy5, with dye swap between samples to eliminate dye bias. The samples were then hybridized to LC Sciences platform. Microarray raw data were normalized using a cyclic LOWESS (Locally-weighted Regression) method, and the mean and co-variance of each probe with a detected signal were calculated. Finally, the data were expressed as the ratio (log_2_ transformed) of the two sets of detected signals, and p-values of the t-test were calculated. The experiment was performed in biological triplicate and the t-test analysis was carried out between NB4 untreated (NB NT 1-3) and NB4 treated with Vorinostat (NB 6h s1-3); p-value ≤ 0.10. In particular, relative fold change for miRNA19a and miRNA19b were 0.32 and 0.36, respectively. Raw and normalized data were uploaded to the NCBI Gene Expression Omnibus (GEO) website and are accessible through GEO Series accession number GSE37374 (http://www.ncbi.nlm.nih.gov/geo/query/acc.cgi?acc=GSE37374).

### miRNA Real-Time PCR

Following RNA extraction, the miRNA fraction was converted into cDNA using miScript Reverse Transcription Kit (Qiagen): 1 µg of RNA was incubated with 1x Buffer, 1xmiScript RT and DEPC-H_2_O for 60 minutes at 37°C and then 5 minutes at 95°C. Subsequently, miRNA Real-Time PCR was performed with QuantiTect SYBR Green PCR Kit (Qiagen), requiring the use of 75 ng of cDNA in presence of 1x QuantiTect SYBR Green PCR Master Mix, miScript Universal Primer and primer specific for miR-19a or miR-19b (Qiagen); RNU6b (Qiagen) specific primer was used to normalize data. The thermal protocol was as follows: 95°C for 15 minutes plus 35 cycles at 94°C for 15 seconds, 58°C for 34 seconds and 70°C for 34 seconds.

### Pre-miR precursor reverse transfection

Pre-miR precursor reverse transfection was performed in NB4 cells with Pre-miR Precursor Starter Kit (Ambion), following the manufacturer’s instructions. Briefly, 5 µl of siPORT Amine Transfection Reagent (Ambion) was diluted into 95 µl of Opti-MEM (Invitrogen) and left for 10 minutes at RT. The reaction mixture was then combined with specific mimic-miRNAs (1:1) at the desired concentration and incubated for 10 minutes at RT; in this case 50 nM mimic-miR-19a and 50 nM mimic-miR-19b were used, or 50 nM mimic-miR-scramble as negative control. This final mixture was added to 4.6 x 10^5^ cells for each point in a 24-well plate.

### BARD1 Real-Time PCR

BARD1 Real-Time PCR was performed using 75 ng of VILO cDNA, 1xSybrgreen PCR Master Mix (Applied Biosystem), 0.5 µM of each primer and sterile H_2_O. The thermal protocol was as follows: 95°C for 15 minutes plus 30 cycles at 95°C for 15 seconds, 60°C for 15 seconds and 72°C for 30 seconds. Primers used: forward exon 8 5’ AGCGTAGGGATGGACCTCTT 3’ (+1691 from ATG) and reverse exon 9 5’ CCATTGAGAATCCCAAGCAT 3’ (+1880 from ATG). GAPDH amplification was used to normalize data.

### Cloning of BARD1 3’UTR

pGL3 control vector was amplified in *E. coli* DH5α (Invitrogen) and extracted with Pure Link Hipure Plasmid Filter Maxiprep Kit (Invitrogen). 1 µg of plasmid with 20U of XbaI restriction enzyme (Biolabs) was then digested for 1 hour and 30 minutes at 37°C, followed by heat inactivation at 65°C for 20 minutes. The digested vector was run on 1% agarose gel to recover the linear vector with QIAEX II Gel Extraction Kit (Qiagen), according to supplier’s instructions. BARD1 3’UTR, both wild-type and mutated, forward and reverse sequences (Bio-Fab) were annealed: 3’UTR wt forward 5’ CATTTCAAATTGAATTTGCACGACATTTCAAATTGAATTGCACGACATTTCAAATTGAATTTGCACG 3’ and reverse 5’ CGTGCAAATTCAATTTGAAATGTCGTGCAAATTCAATTTGAAATGTCGTGCAAATTCAATTTGAAATG 3’; 3’UTR mut forward 5’ CACCCCAAATTGAATTTGCACGACACCCCAAATTGAATTTGCACGACACCCCAAATTGAATTTGCACG and reverse 5’ CGTGCAAATTCAATTTGGGGTGTCGTGCAAATTCAATTTGGGGTGTCGTGCAAATTCAATTTGG GGTG.

 Each sequence was designed with sticky ends for XbaI enzyme (TCTAGA) followed by a unique site of digestion recognized by BlpI enzyme (GCTGAGC) and 3’UTR BARD1 sequence repeated three times. The two fragments at 1 µg/µl concentration were mixed by 1:1 balance and incubated at 90°C for 5 minutes, 37°C for 30 minutes and 4°C for 10 minutes. The ligation reaction was then performed: 100 ng of vector were mixed with 2000 U of T4 ligase enzyme, 1 µl of 30% PEG 8000, 3’UTR insert, 1x ligase buffer and DEPC-treated H_2_O; the quantity of insert for use was determined by the formula: [(ng of vector x kb insert) / kb vector] x 20/1; the ligase reaction was carried out at 16°C overnight. The following day 10 µl of ligase product was used to transform *E. coli* DH5α and finally pGL3-3’UTR-BARD1 wt and mut vectors were recovered with Pure Link Hipure Plasmid Filter Maxiprep Kit (Invitrogen). 

### Transfection

pGL3-3’UTR-BARD1 wt and mut vectors were transfected into HeLa cells using Lipofectamine™ 2000 Transfection Reagent (Invitrogen), following supplier’s instructions. 1 µg of pGL3-3’UTR-BARD1 plasmid plus 200 nM mimic-miR-19a, mimic-miR-19b or mimic-miR-scramble were used.1 µg of pMAX-GFP vector was transfected in all the samples to normalize data.

### Luciferase assay

Luciferase activity was measured after 24 hours and 48 hours from pGL3-3’UTR-Bard1 transfection. HeLa cells were incubated with Luciferase Cell Culture Lysis 5x Reagent (Promega) for 1 hour at RT with gentle shaking, and then the cell lysate was transferred into a 96-well white plate (Nunclon) to read GFP fluorescence values (λexc = 485 nm; λemi = 520 nm); 100 µl of Luciferase Assay Reagent (Promega) were then added and luminescence values were quantified. All of these measurements were calculated using TECAN infinite M200 station. Fluorescence values were used to normalize data, expressed as a percentage of luminescence on percentage of fluorescence ratio. 

### pCMV-MIR stable transfection

pCMV-MIR vectors (OriGene) encoding for miR-19a or miR-19b and the empty vector were first amplified in *E. coli* DH5α and plasmids were extracted with PureLinkHipure Plasmid Filter Maxiprep Kit (Invitrogen). Subsequently, 1 µg of each vector was transfected into U937 cells using AMAXA Nucleofector (Lonza), according to supplier’s instructions. 48 hours after transfection, the percentage of GFP- positive cells was determined by FACS analysis; the cells were then placed in a 96-well plate in presence of 500 µg/ml G418 (Gibco) to isolate the positive clones. 

### Cell cycle analysis

Cells were collected by centrifugation at 1200 rpm for 5 minutes and then resuspended in 500 µL of a hypotonic buffer composed of 0.1% NP-40, 0.1% sodium citrate, 50 µg/ml propidium iodide (Sigma Aldrich), RNAse A. The samples were then incubated in the dark for 30 minutes. Analysis was performed by FACS-Calibur (Becton Dickinson) using Cell Quest Pro software (Becton Dickinson) and ModFit LT version 3 software (Verity). Experiments were performed in duplicate. 

### Caspase assay

Caspase activity was detected within whole living cells using FLICA Apoptosis Detection Kits (B-Bridge). Green FAM-LETD-FMK and red SR-LEHD-FMK substrates were used for caspase-8 and caspase-9 respectively. Cells untreated or treated with 5 µM Vorinostat for 24 hours were washed twice in cold PBS and incubated for 1 hour in ice with the corresponding substrates, as recommended by suppliers. Washing step and fluorescence analysis were then performed using Cell Quest Pro software (Becton Dickinson) applied to FACS-Calibur (Becton Dickinson). Experiments were performed in duplicate. 

### siBARD1 transfection

Specific BARD1 fluorescent siRNA (Hs_BARD1_7 and Hs_BARD1_5 FlexiTubesiRNA, Qiagen) were transfected into U937 cells with AMAXA Nucleofector, according to the supplier’s instructions. The total amount of siRNA used was 2 µg. 24 hours after transfection cells were treated with 5µM Vorinostat for additional 24 hours. Finally, 48 hours after transfection cell viability was evaluated by FACS-Calibur (Becton Dickinson) and Cell Quest Pro software (Becton Dickinson), after PI staining of dead cells. The percentage of green fluorescent cells was also measured by FACS analysis. AllStars negative siRNA was used as negative control. 

### BARD1 expression analysis *in vivo* (human)

BARD1 expression was assessed by RT-PCR after RNA extraction and cDNA conversion as previously described. Experiments were performed in samples from patients treated as in [38].

### Expression and detection of biotinylated BARD1 isoforms in human cell culture

The PCR fragment BARD1 corresponding to full length BARD1 CDS or BARD1ω15’UTR and CDS were cloned into pcDNA3.1 (+) vector (Invitrogen) (forward primer annealing sequence ATCACAATAGACTTTCCAGTC). Their 3’ ends were fused in-frame with a sequence encoding the 30-amino acid BLRP motif, which is specifically biotinylated by bacterial BirA biotin ligase [39]. To express biotinylated BARD1 fusions HEK293T cells were co-transfected with these constructs and BirA also cloned in pcDNA3.1(+). Recombinant proteins were visualized with streptavidin conjugated to DyLight 488 and observed using fluorescent microscope.

### Statistical analysis of the distribution of nuclei size

HEK293T cells were transfected with Omega-biotin expressing construct as described in corresponding “Materials and methods” section. The transfected cells were fixed for microscopic examination. Biotinylated proteins were visualized as described and counterstained with DAPI to visualize DNA. The images of DAPI and biotinylated proteins staining taken at 20x magnifications were used for the quantification of nuclei size. The size of the nuclei was quantified for the random samples (no less than 100 nuclei each) of the cells not visibly expressing or visibly expressing recombinant protein. ImageJ 1.46r software (http://imagej.nih.gov/ij) was used to quantify the surface of the nuclei images. The distribution of the obtained values was analyzed using Peltier Tech Excel Box and Whisker Plot (Box Plot) Utility (http://peltiertech.com) using Excel quartiles calculation and is represented as Box and whisker quartile chart. The inner quartiles of each sample are represented by grey boxes, separated at the median by a black line. The lower and upper quartiles are represented by whiskers. The average of each sample is represented by a diamond marker. The close and far outliers are shown as red crosses and red circles correspondingly. Unpaired two tailed Student T-test was used to calculate p-value characterizing the difference between these two samples.

## Results

### BARD1 isoform(s) comprising exons 6 to 11 are highly expressed in human AML ex vivo samples

**Figure 1:**
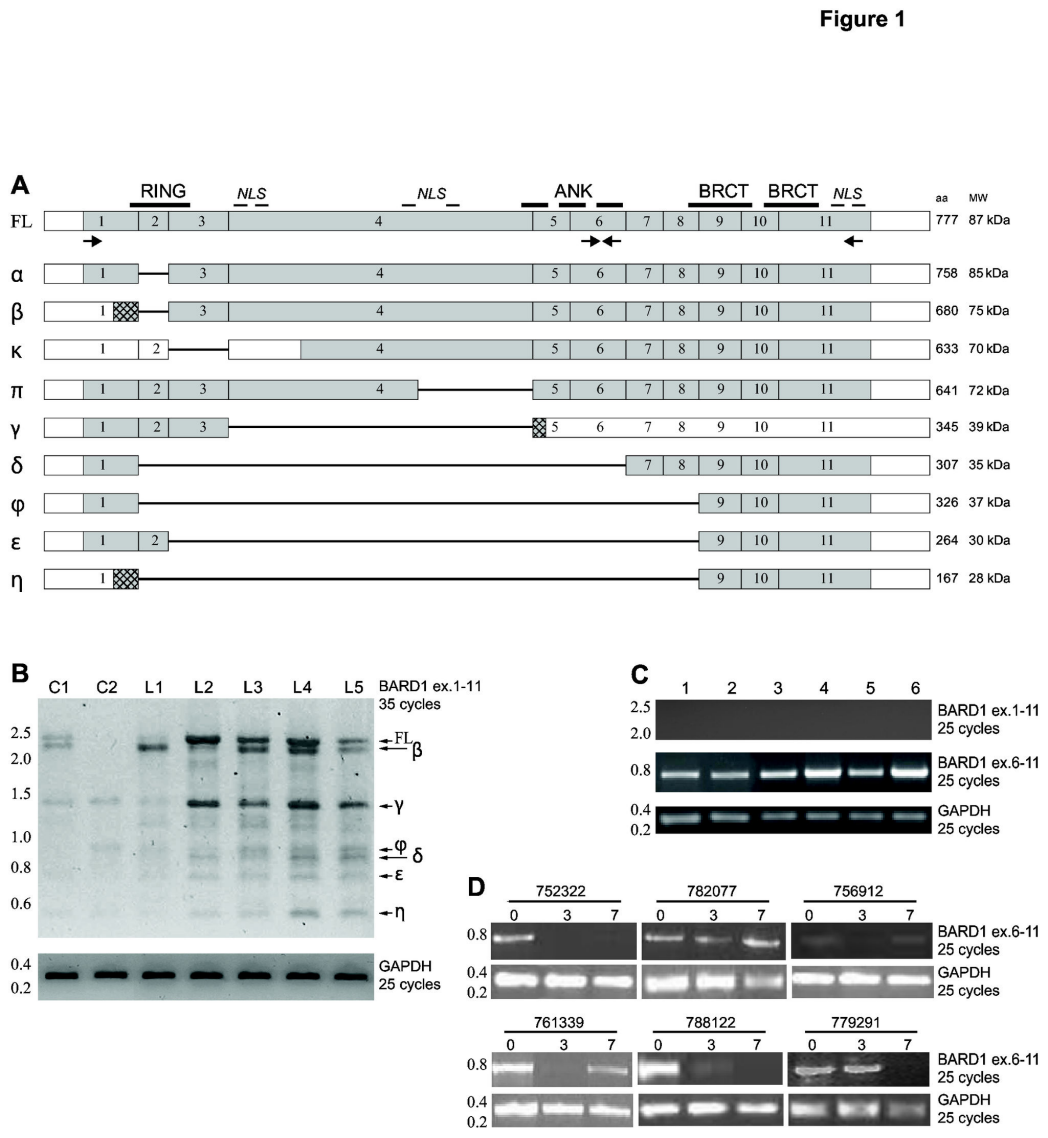
Truncated BARD1 isoforms are highly expressed in human AML *ex* *vivo* and *in* *vivo* samples. (*A*) Schematic diagram of exon structure of full length (FL) BARD1 and deletion-bearing BARD1 isoforms. Approximate positions of BARD1 protein motifs, RING finger, ANKyring repeats, BRCT domains, and nuclear localization signals (NLS) are indicated above FL BARD1 structure, primers used for RT-PCR are indicated below. Exon structure of BARD1 isoforms α, β, κ, γ, ϕ, δ, ε, and η are aligned with FL BARD1. Grey boxes indicate protein coding, empty boxes non-coding regions, lines represent missing exons. Number of encoded amino acids (aa) and calculated molecular weight (MW) are indicated on the right side. (*B*) BARD1 RT-PCR using primers in exons 1 and 11 in *ex* *vivo* samples. With 35 cycles of PCR amplification, only little expression of FL BARD1 and isoforms is detected in blood cells of healthy controls (indicated as C), but all forms are present in leukemia patients (L1-L5). In comparison 25 cycles of amplification of GAPDH is shown. (*C*) RT-PCR for amplification of BARD1 exons 6 to 11 in *ex* *vivo* AML samples (see also Table 1). With 25 cycles of amplification using primers in exons 1 and 11, no PCR products are detected, however, primers in exons 6 and 11 show fragments in all samples, as it is the case for PCR of GAPDH. (*D*) BARD1 RT-PCR in patient’s samples at different time points of in vivo treatment: D0 (pre-treatment), D3 (3 days of Vorinostat treatment) and D7 (3 days of Vorinostat + 4 days of Ara-C and Idarubicin treatment. GAPDH represents mRNA concentration and loading control.

We firstly evaluated BARD1 expression levels in human AML *ex vivo* blasts. Given that BARD1 mRNAs might be present as different isoforms (Figure 1A) [3], we used two different pairs of primers: exons 1 to 11 and exons 6 to 11. The former is able to amplify full length (FL) α, β, κ, π, φ, δ, ε and η BARD1 isoforms, which contain the first and last exons but can be distinguished by deletion of internal exons (different lengths). The latter amplifies FL α, β, κ, π isoforms, which contain exon 6 to exon 11 fragment but have different transcription start sites (TSS) [3]. RT-PCR analysis clearly indicated the presence of BARD1 in all patient samples. In particular, isoforms containing exon 6 to exon 11 were strongly expressed and more abundant in comparison with FL and other long BARD1 isoforms, which appear weakly expressed in all AML samples under identical conditions for amplification (Figure **1B-C** and Table **1**). In contrast, peripheral blood cells from healthy individuals showed expression of FL BARD1 and several smaller isoforms (Figure **1B**). 

**Table 1:** Characteristics of AML samples.

**Patient n°**	**Age**	**Diagnosis**	**FAB**	**Cyto**	**Phenotype**
P_1	67	APL	M3	t(15;17)	DR-, CD34-, CD13+, CD33+, CD117+, CD45RA+, CD45RO-
P_2		AML			
P_3		AML		46, XX	CD13+, CD33+, CD24+, CD11c, CD16+/-
P_4	30	AML	M2		(gate ≈45%) CD34+, CD13+, HLA-, DR+, CD19+, CD45RA+, CD71+, CD117+, MPO+
P_5	58	AML	M2		CD33+, CD13-, CD11c+, CD11b+/-, CD117+/-, CD34+/-, DR+, MPO-
P_6	63	AML	M1		CD13+/-, CD33+, CD117+/-, CD14+/-, CD34+/-, CD45+/-

### Modulation of BARD1 expression in patients undergoing Vorinostat treatment

By RT-PCR we measured BARD1 isoform exon 6 to 11 expression in patients undergoing Vorinostat anti-AML therapy followed by Ara-C and Idarubicin administration [38]. These experiments revealed that BARD1 isoforms are often present in these patients and, interestingly, their expression could be reduced by the clinical protocol, in particular after the 3 days of treatment with the HDACi Vorinostat (Figure **1D** and Table **2**, **3**) in some of them. These data were confirmed in AML patient’s samples *ex vivo* (Table **3**). These results seem to suggest that not all AML patients express BARD1, the majority of them displaying a basal down-regulation of the FL BARD1 form in favor of shorter isoforms or no BARD1 expression. In addition, some patients display a decreased expression after treatment with Vorinostat (Figure **1D**). Based on these findings, a classification of patients based on BARD1 isoform expression levels could be possible. 

**Table 2:** Characteristics of patients enrolled in the Vorinostat-based clinical trial.

**Patient n°**	**Sex**	**Age**	**% Blasts PB**	**% Blasts BM**	**FAB**	**Cyto**	**Mol Bio**	**Flow cytometry**
779291	F	56	90	62	M2	46, XX	NPM1	CD13, CD15, CD33, CD117, CD38, CD64
788122	F	22	46	91	M0	Complex	Neg	CD13, CD33, CD117, CD15, CD64
756912	F	21	96	86	M2	46, XX	FLT3+	CD117, ndim CD64, CD38, CD33, CD13
752322	M	55		26	Unk	−Y	FLT3, ITD	CD13, CD2, CD117, CD33, CD34, HLADR
782077	F	54	18	38	M5	Complex	Neg	ND
761339	M	48	2	24	RAR EB-T	−5, −7	ND	CD13, CD15+/-, CD117, CD34, CD38

**Table 3:** Characteristics of AML patient’s samples and BARD1 expression analysis.

**Sample**	**AML**	**Bard 1 isoform expression**
222		+
251	M1, NPM1+, FLT3+	+
182	M4	+
116	M1	-
204		+
183	M2, 47 XY+der(1)-2-7+	-
257	M4, FLT3+ (ITD); mNPM	+
265		+
270	M2	+
272	M1, complex	-
252	M4	-
211	M1	+
225	AML	+

### Truncated BARD1 isoform(s) are also expressed in human AML cell lines and their expression is modulated by HDACi treatment

**Figure 2:**
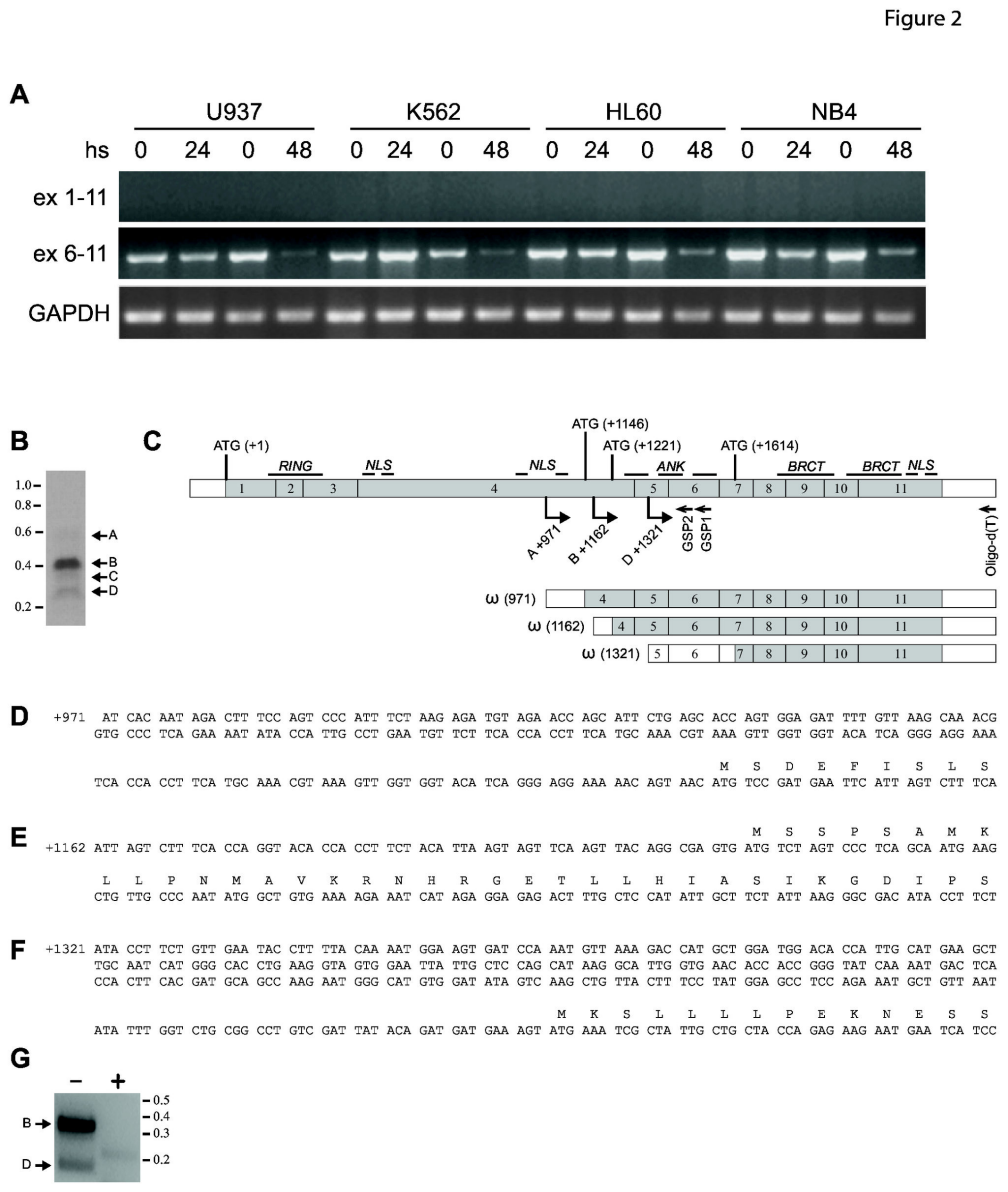
Truncated BARD1 isoform(s) are expressed in human AML cell lines and their expression is modulated by HDACi treatment. (*A*) BARD1 expression levels in U937, K562, HL60 and NB4 cells, untreated or treated with Vorinostat 5 µM for the indicated times. GAPDH represents mRNA concentration and loading control. (*B*) Characterization of truncated BARD1 isoform(s). 5’ Race was performed to determine the 5’ end(s) of upregulated isoforms of BARD1 comprising exons 6 to 11. 5’Race performed with mRNA of NB4 cells and amplified with nested gene specific primers (GSP) as indicated in (C) exhibited three isoforms with different 5’ends, labeled A, B, and D. Fragment C was an unrelated sequence. (C) Schematic representation of truncated BARD1 isoforms identified by 5’RACE PCR. Locations of primers and GSPs and transcription start sites are indicated, as well as structure of 5’ truncated mRNA, termed ω isoforms. (*D*, E, F) Nucleotide sequence of 5’ends of ω isoforms and their deduced amino acid sequences. (G) 5’Race of untreated (-) or SAHA (+) treated NB4 cells clearly shows repression of amplicons A and D, corresponding to ω(1162) and ω(1321) isoforms.

Based on the *ex vivo* data, we decided to exploit the de-silencing activity of the epigenetic modifiers HDACis in AML cell lines to obtain the re-expression of long BARD1 isoforms, including the FL. To this end, we determined the BARD1 expression profile in four different human AML cell lines (NB4, U937, K562 and HL60) together with cell lines of different origin. FL and other long isoforms were weakly, if at all, expressed (Figures **2** and **S1**), corroborating the results obtained *ex vivo*. Interestingly, the specific isoform(s) containing exons 6 to 11 were strongly expressed in AML cell lines (Figure **2**). We then stimulated these AML cells for 24 or 48 hours with the well-known HDACi Vorinostat, used at 5 µM concentration. A down-regulation of the truncated (exons 6 to 11) BARD1 isoforms by Vorinostat was observed, as well as longer BARD1 isoforms (Figure **2A, S2 and S3**). This effect on BARD1 expression was time-dependent; NB4 cells proved particularly sensitive to Vorinostat, displaying BARD1 down-regulation as early as 6 hours after treatment (Figure **S2**). In fact Vorinostat was more effective than Entinostat, MC 1568 and ST 80 (Figure **S2C**), which strongly suggests that both class I & II HDACs may play a role in BARD1 down-regulation.

### Characterization of truncated BARD1isoform(s)

RT-PCR experiments showed that truncated isoform(s) of BARD1 are strongly expressed in AML cells compared to FL BARD1 and other internal deletion-bearing BARD1 isoforms. To identify these specific BARD1 isoform(s) containing the exon 6 to 11 sequences, 5’ RACE PCR was performed on RNA samples from NB4 cells. We characterized three BARD1 isoforms (Figure **2B-G**), two of which starting at the end of exon 4 (+971 and +1162 from ATG) and the third at exon 5 (+1321 from ATG). We subsequently termed these N-terminally truncated isoforms ω1, 2, and 3. These isoforms seem particularly prominent in NB4 cells. The protein translated from isoforms starting in exon 4 would have a size of 395 amino acids or 44.39 kDa, comprise the ankyrin repeats and BRCT domains, but not the RING finger, and the one starting in exon 5 would code for 239 amino acids or 27.3 kD, comprising only the BRCT domains (Figure **2B-G**).

### BARD1 expression is also modulated by Vorinostat treatment in other cancer cell lines

To determine whether Vorinostat-induced BARD1 down-regulation was an exclusive event of human AML cells, we evaluated BARD1 expression levels in other human cancer models, such as MCF7 breast cancer cells, HeLa cervical cancer cells and Kelly neuroblastoma cells. The time-dependent reduction of these truncated BARD1 isoform(s) after Vorinostat stimulation was also observed in Kelly and MCF7 cells, but not in HeLa cells, suggesting that a restricted panel of cancer cells displays BARD1 deregulation. Interestingly, FL BARD1 is weakly expressed and affected by Vorinostat (Figure **S3**).

### Vorinostat induces overexpression of different miRNAs in human AML cell lines

**Figure 3:**
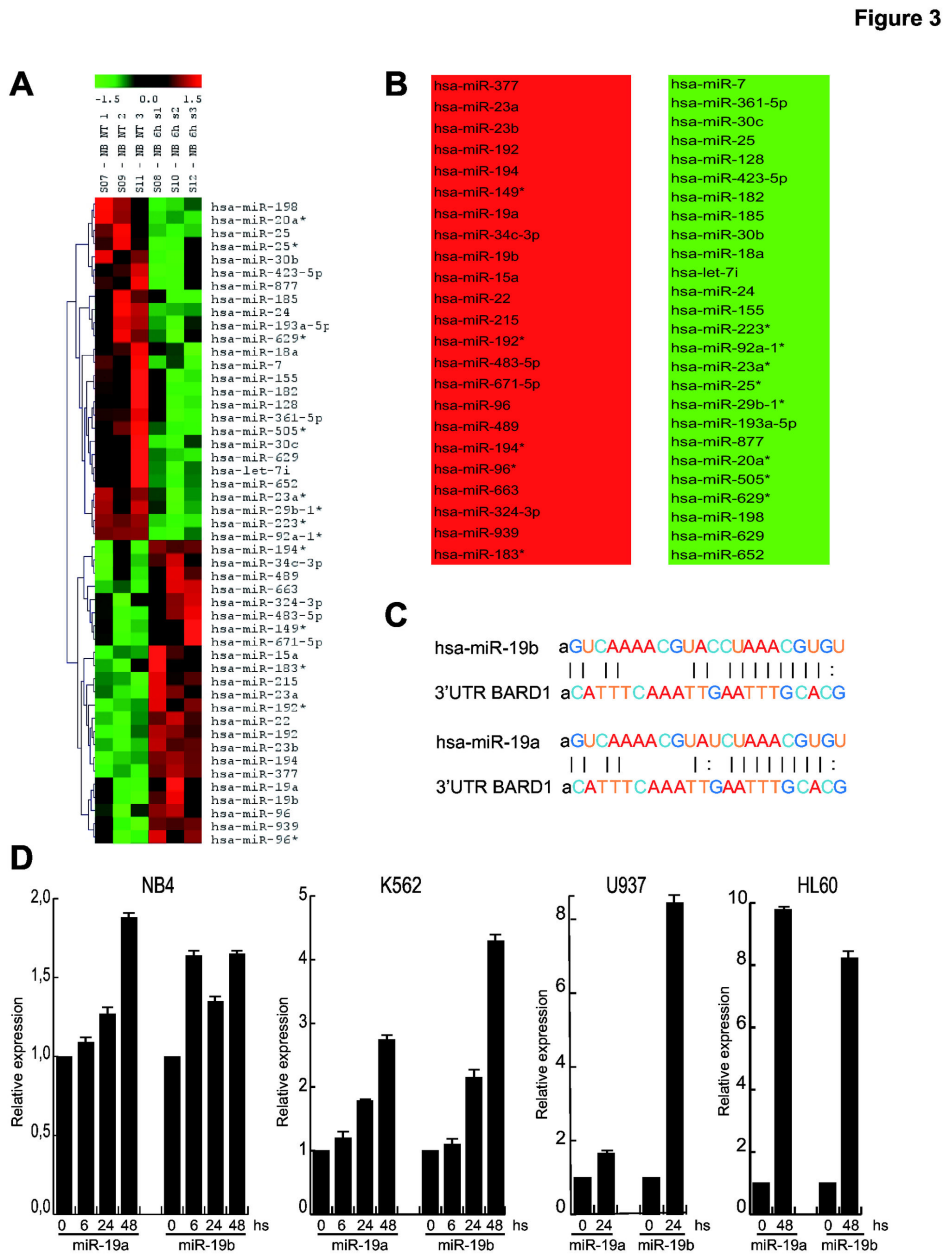
Vorinostat induces overexpression of different miRNAs in human AML cell lines. (*A*) Heat-map of miRNAs altered in NB4 cells after treatment with Vorinostat (5 µM) for 6 h (s); the experiment was performed in biological triplicate and the t-test analysis was carried out between NB4 untreated (NB NT 1-3) and NB4 treated with Vorinostat (NB 6h s1-3); p-value ≤ 0.10. (*B*) Table of miRNAs up-regulated (in red) and down-regulated (in green) after Vorinostat treatment; p-value ≤ 0.10. (*C*) Annealing of miR-19a and miR-19b to BARD1 3’UTR. (*D*) Real-Time PCR for miR-19a and miR-19b in NB4, K562, U937 and HL60 cells after Vorinostat at a concentration of 5 µM. Data show the mean value of three parallel experiments with error bars showing the standard deviations on top of each column.

We hypothesized that cell type specific BARD1 down-regulation after Vorinostat stimulation might be mediated by miRNA action. We performed miRNA microarray expression analysis using NB4 cells, untreated or treated with Vorinostat at a concentration of 5 µM for 6 hours, to identify miRNAs exhibiting modulated expression. A close group of miRNAs was altered after Vorinostat treatment, among which 23 miRNAs was shown to be overexpressed in these cells (Figure **3A and 3B**). By computational prediction of miRNA target gene using microRNA.org website, we focused on two miRNAs that may potentially regulate BARD1 expression by the binding to its 3’UTR: miR-19a and miR-19b (Figure **3C**).

The overexpression of miR-19a and miR-19b in NB4 cells was confirmed by qPCR in NB4, K562, U937 and HL60 cells (Figure **3D**). We concluded that the decrease of BARD1 mRNA observed by Vorinostat treatment could involve miR-19a and miR-19b.

### BARD1 is a target of miR-19a and miR-19b

**Figure 4:**
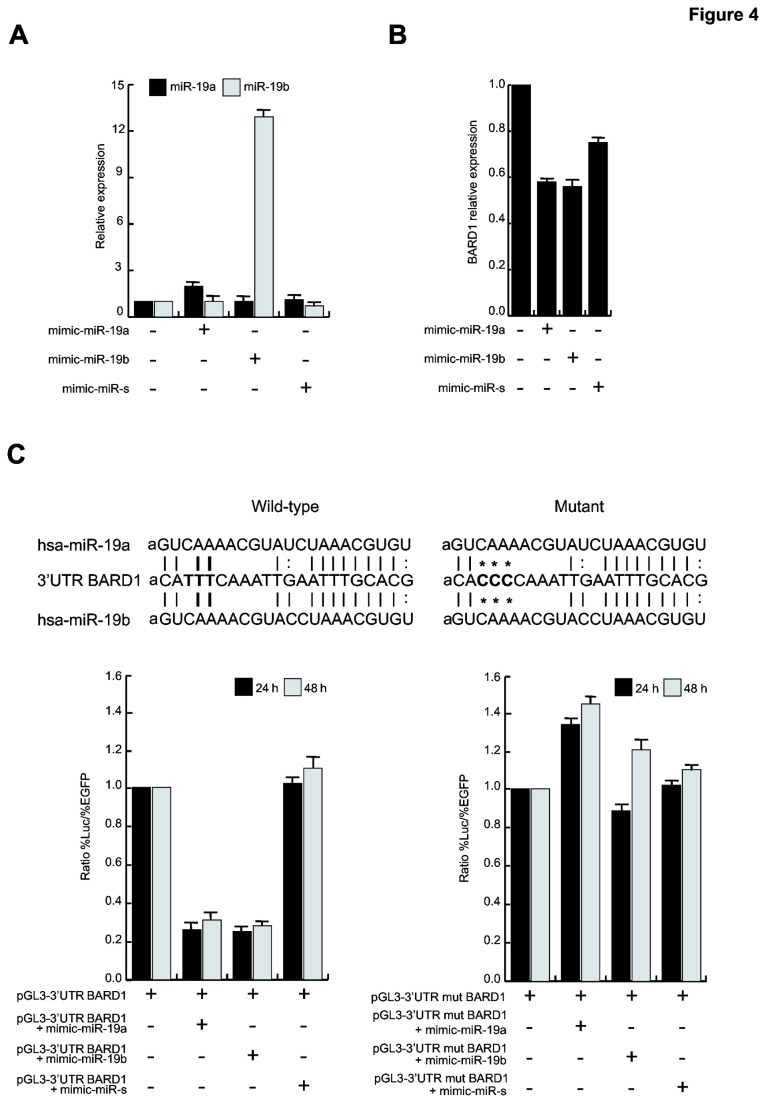
BARD1 is the target of miR-19a and miR-19b. (*A*) Real-Time PCR for mir-19a and miR-19b in NB4 cells transfected with mimic-miR-19a, mimic-miR-19b or mimic-miR-scramble (miR-s). Data show the mean value of three parallel experiments with error bars showing the standard deviations on top of each column. (*B*) BARD1 Real-Time PCR in NB4 cells transfected with mimic-miR-19a, mimic-miR-19b or mimic-miR-scramble. Data show the mean value of three parallel experiments with error bars showing the standard deviations on top of each column. (*C*) Luciferase assay in HeLa cells after transfection with 1 µg pGL3-3’UTR-BARD1 wild-type (left) and mutated (right) vectors plus mimic-miR-19a/b or scramble at a concentration of 200 nM. Data show the mean value of three parallel experiments with error bars showing the standard deviations on top of each column. Annealing of miR-19a and miR-19b to BARD1 3’UTR: wild type and mutated BARD1 3’UTR are schematized.

To confirm that BARD1 is a target of miR-19a and miR-19b, we transfected NB4 cells with mimic-miR-19a and mimic-miR-19b. As expected, Real-Time PCR analysis showed an increment of hsa-miR-19a and hsa-miR-19b expression 48 hours after transfection (Figure **4A**) compared to the untreated control and the mimic-miR-scramble control. Corroborating our hypothesis of regulation of BARD1 mRNAs by miRs, miR-19a and miR-19b overexpression led to BARD1 reduction (Figure **4B**). These observations demonstrate that BARD1 is a target of miR-19a and miR-19b.

### miR-19a and miR-19b directly bind BARD1 3’UTR reducing its expression levels

To further demonstrate the direct binding of miR-19a and miR-19b to the 3’UTR of BARD1, we cloned the BARD1 3’UTR region into the pGL3 vector, downstream of the luciferase reporter gene. We then transfected HeLa cells with pGL3-3’UTR-BARD1 vector plus the mimic-miRs, and measured luciferase activity 24 and 48 hours after transfection. The luciferase assay clearly showed that miR-19a and miR-19b can directly bind to the 3’UTR of BARD1, decreasing luciferin oxidation and light development (Figure **4C** **, left**), compared to the HeLa control cells transfected only with pGL3-3’UTR-BARD1 vector and the mimic-miR-scramble control. Notably, BARD1 3’UTR mutated form was not able to bind the two miRs and, consequently, to decrease the luciferase signal (Figure **4C** **, right**), thus strongly strengthening the results.

### miR-19a and miR-19b overexpression leads to increased sensitivity to Vorinostat treatment

**Figure 5:**
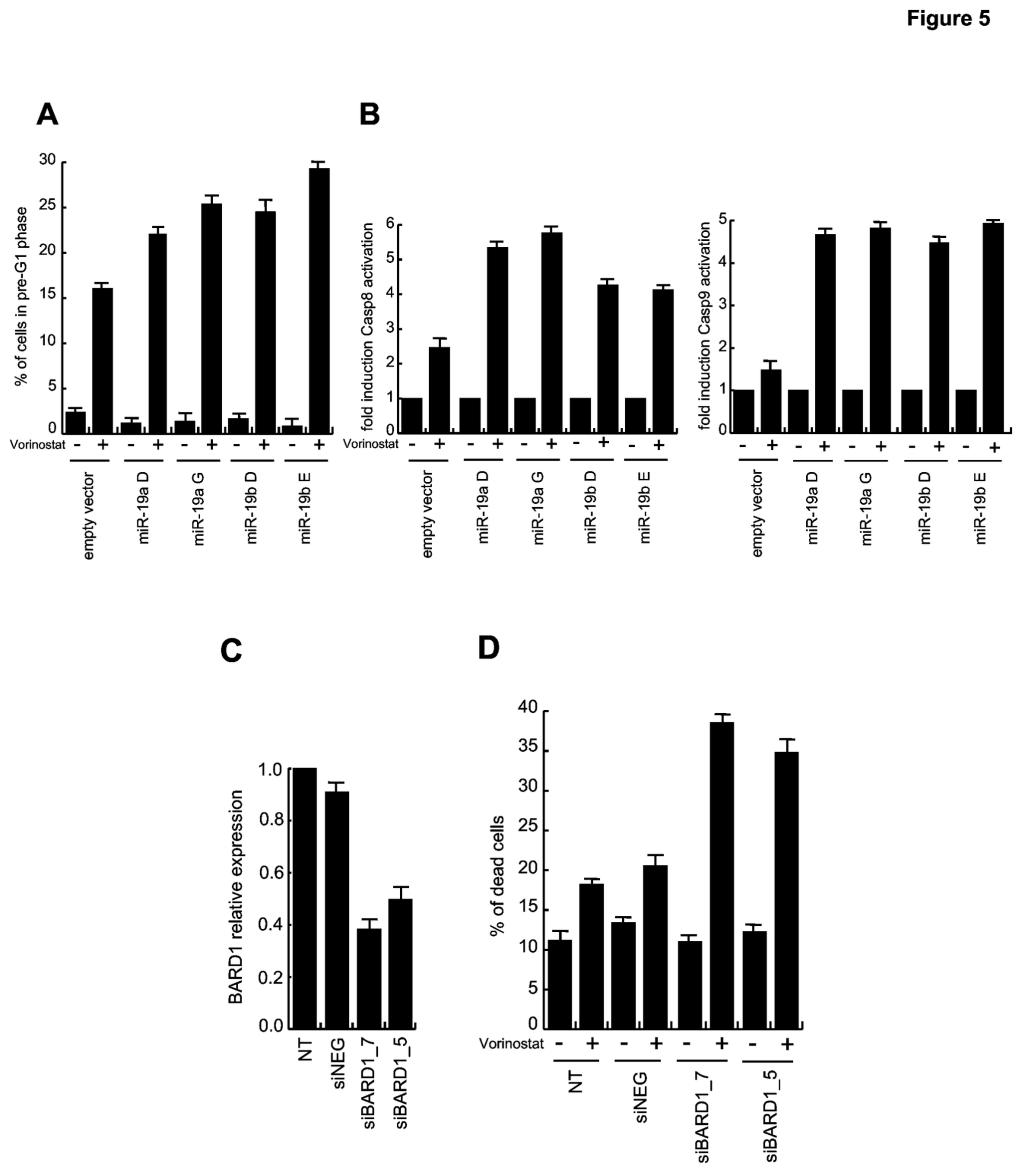
Modulation of miR-19a, miR-19b and BARD1increases mortality of U937 cells after Vorinostat treatment. (*A*) Cell death analysis in U937-mir cells after 24 h treatment with Vorinostat (5 µM). Data show the mean value of three parallel experiments with error bars showing the standard deviations on top of each column. (B) Caspase-8 and -9 activation in U937-mir cells treated for 24 h with Vorinostat (5 µM). Data show the mean value of three parallel experiments with error bars showing the standard deviations on top of each column. (*C*) BARD1 expression levels measured by Real-Time PCR in U937 cells transfected with specific siRNAs as indicated. Data show the mean value of three parallel experiments with error bars showing the standard deviations on top of each column. (*D*) Analysis of cell death by PI incorporation after 24 h of Vorinostat treatment (5 µM) in U937 siRNA-transfected cells. Data show the mean value of three parallel experiments with error bars showing the standard deviations on top of each column.

To clarify the biological functions of miR-19a and miR-19b, stable transfection of pCMV-MIR-19a, pCMV-MIR-19b into U937 cells was performed. Clones were screened for overexpression of the two miRNAs and clones U937-miR-19a D and G and U937-miR-19b D and E were selected for further investigation (Figure **S4**). Upon treatment with Vorinostat, U937-miR-19a and U937-miR-19b cells showed a higher percentage of cell death in comparison with the negative control (Figure **5A**). Caspase assays in U937-MIR cells further suggested that in U937-miR-19a and U937-miR-19b cells Vorinostat treatment induced stronger caspase-8 and, in particular, caspase-9 activation (Figure **5B**).

### BARD1 silencing increases mortality of U937 cells after Vorinostat treatment

To understand whether BARD1ω1isoform expression contributes to AML maintenance, we performed transfection experiments with specific BARD1 siRNAs, followed by administration of 5 µM Vorinostat for 24 hours. Cells with lower BARD1ω1 isoform expression (Figure **5C and D**) displayed a higher percentage of cell death, thus corroborating the involvement of BARD1 isoforms in inhibiting apoptosis pathways.

### BARD1 Ω1 inhibits apoptosis

**Figure 6:**
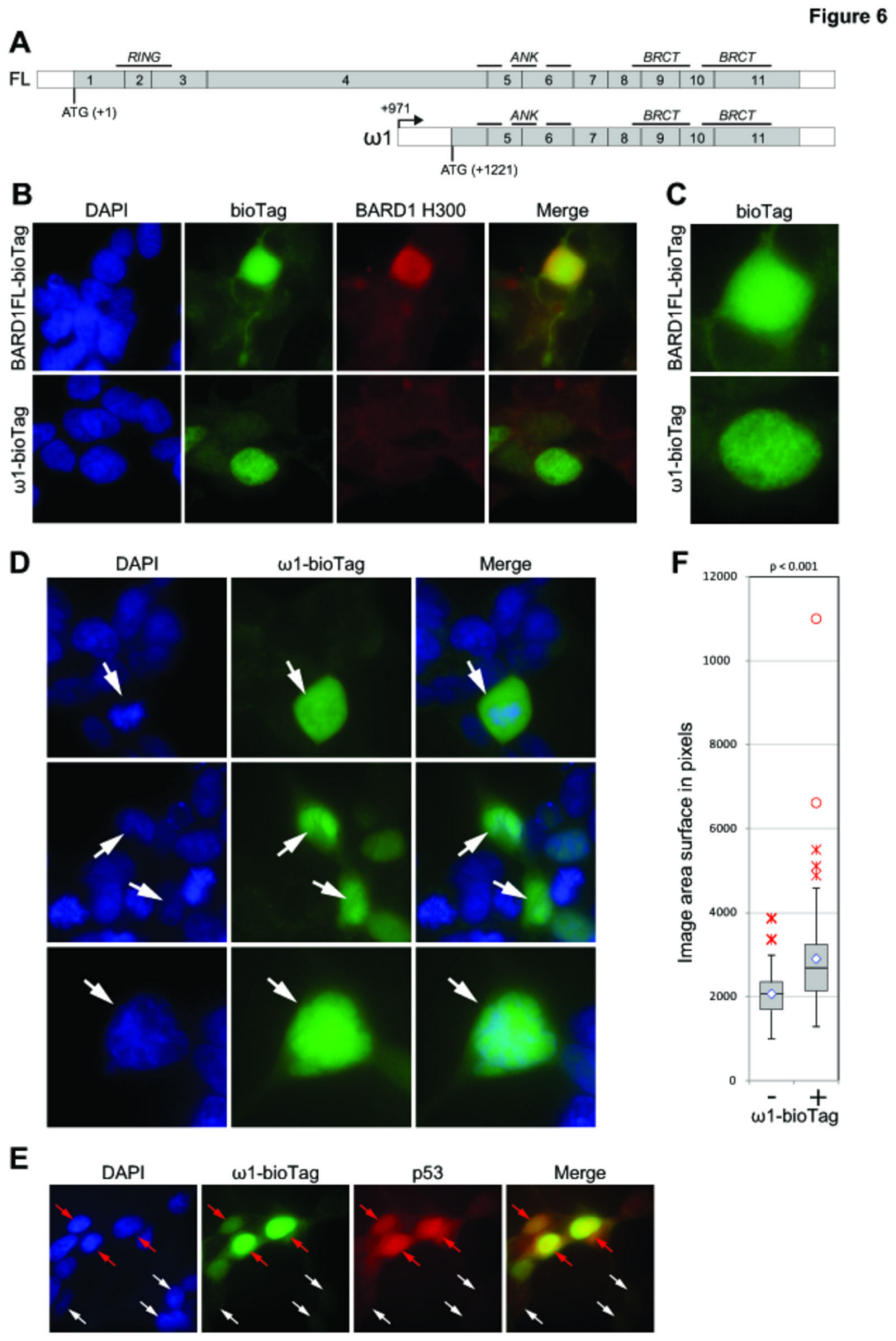
Visualization of BARD1ω fused to the biotin tag in HEK293T cells. (A) Intron structure and protein domain scheme of FL BARD1 and BARD1ω1 mRNA. Non-coding sequences are shown as a black line; protein-coding sequences are shown as grey bars. (B) BARD1 full length and BARD1ω1 fused to N-terminal biotin tag (ω1-bioTag) were visualized with streptavidin conjugated to DyLight 488 at 20x magnification. The cells were co-stained with Bethylanti-BARD1 H300 antibody recognizing C-terminal fragment of BARD1. As expected, the antibody recognizes BARD1FL-bioTag, but not BARD1ω1-bioTag protein. Non-transfected cells show weak peri-nuclear staining due to endogenously biotinylated proteins localized mostly in mitochondria. Note predominantly nuclear localization of BARD1ω1-bioTag. (C) The distribution of recombinant BARD1 full length and BARD1ω1 in the interphase nucleus. (D) The distribution of BARD1ω1 and phenotype of the cells expressing BARD1ω1 (all panels are shown at 40x magnification). Upper panel: the distribution of BARD1ω1-bioTag in the anaphase nucleus (white arrow). Note that BARD1ω1-bioTag is not co-localized with DNA and enriches the nucleoplasm. Middle panel: the cells expressing BARD1ω1-bioTag are frequently appear multi-nuclei (marked with arrows). Lower panel: nuclei of the cells expressing BARD1ω1-bioTag are significantly bigger than the cells with undetectable levels of the fusion protein but show the same DNA density (marked with arrows). (E) p53 content is higher in the cells overexpressing ω1-bioTag (red arrows) then in the cells with low level of recombinant protein (white arrows). We hypothesize that ω1-bioTag may bind p53 protecting it from degradation and tethering it out from its normal localization. (F) Over-expression of Omega affects the size of the nuclei. The box-plot represents the distribution of the size of nuclei of the cells not visibly expressing (-) or expressing (+) BARD1 omega-biotin. The size of the nuclei was quantified using the microscopic image of DAPI stained cells taken at 20x magnification. The sample size is no less than 100. The inner quartiles of each sample are represented by grey boxes, separated at the median by a black line. The lower and upper quartiles are represented by whiskers. The average of each sample is represented by a diamond marker. The close and far outliers are shown as red crosses and red circles correspondingly. Unpaired two tailed Student T-test demonstrates that this distributions are significantly different with the p-value < 0.001.

To determine how BARD1ω1 isoform expression counteracts the pro-apoptotic signal of Vorinostat treatment, we investigated its intracellular localization. A biotin tagged form of BARD1ω1 was cloned and expressed in HEK-293 cells (Figure **6** and Figure **S5**). BARD1 Ω1 expressing cells were detected with streptavidin interaction. Comparison of GFP-FL BARD1 expression and Bio-BARD1ω1 showed that both localized primarily to the nucleus, although in a different manner. GFP-FL BARD1 localizes to nuclear speckles, whereas Bio-BARD1ω1 showed a homogeneous distribution, suggesting a different interaction with protein binding partners (Figure **6A and B**). 

We also investigated whether Bio-BARD1ω1 cells showed defects in mitosis, as was reported for other BARD1 isoforms [6,8]. Indeed Bio-BARD1ω1 expressing cells showed a significantly increased number of aberrant mitotic figures, such as aberrant chromosome alignment at the metaphase and anaphase state and abnormally increased size of their nucleus (Figure **6C-E**). 

To investigate the observed increase of apoptosis in cells that were repressed for BARD1ω1, and considering that FL BARD1 was reported to promote p53-dependent apoptosis [12,14,15], we co-stained cells with anti-p53 antibodies. Indeed p53 signal was massively increased in Bio-BARD1ω1 expressing cells, as compared to un-transfected cells (Figure **6F**). Note that both exogenous and endogenous expression of ω isoforms are shown in Figure **S6A-B**. Since the intracellular distribution of Bio-BARD1ω1 is different from FL BARD1, these observations suggest that Bio-BARD1ω1 stabilizes and sequesters p53, but does not promote p53 dependent apoptosis. 

## Discussion

In the present manuscript, we identified three BARD1 isoforms of potential interest as cancer markers in AML. The substitution of FL BARD1, weakly (if at all) expressed in *ex vivo* AML blasts and human AML cell lines, with truncated BARD1 isoforms suggests that this process causes or contributes to leukemogenesis. Thus, the three overexpressed truncated BARD1 mRNA isoforms, together with FL BARD1 down-regulation, might be *bona fide* candidates as diagnostic tumor markers. Consistently, FL BARD1 was observed to be down-regulated not only in leukemia [18], but also in colon cancer patients [4] and NSCLC samples [5]. It is tempting to speculate that frequent loss or reduced expression of FL BARD1 might also be a poor prognosis factor. Moreover, some BARD1 isoforms were expressed in both cancer and adjacent healthy tissues [5], leading to the conclusion that isoform expression could be involved in the initiation of tumorigenesis, thus ‘marking’ an early step of cancer progression. 

The oncogenic potential of truncated BARD1 isoforms might be related to their lack of some important domains, in particular the RING N-terminal domain, consistent with the loss of BARD1 tumor-suppressor functions. Moreover, expression of truncated BARD1 isoforms has been correlated with poor prognostic factors in breast, ovarian and lung cancer, suggesting a pro-tumorigenic function of the isoforms [5]. In particular, the truncated BARD1 isoforms described here are similar, but not identical, to those described previously [3] devoid of the RING domain and BRCA1interaction, which result in altered ubiquitination activity on target proteins and perturbation of cell cycle progression [2].

We demonstrate that BARD1 is a target of miR-19a and miR-19b, two miRNAs belonging to the miR-17-92 cluster, located on chromosome 13. 

The miR-17-92 cluster is known as ‘oncomir-1’, since its encoded miRNAs are often amplified in lymphomas and several solid tumors, including breast, lung and colon cancers [40]. miR-17-92 cluster appears to be able, together with *c-myc*, to induce B-cell lymphoma development in a mouse model [8,9] and to increase proliferation of normal and malignant lung cells, while inhibiting their differentiation [41]. miR-19b, especially, seems to play a crucial role in inducing carcinogenesis. However, apart from its ability to increase cell proliferation and to exert an anti-apoptotic effect by targeting death-promoting proteins opposed to PI3K (Phosphatidyl-Inositol-3-kinase)/Akt pathway in Acute Lymphoid Leukemia (ALL) [42], the exact mechanism responsible for its oncogenicity remains unknown. On the other hand, there is also evidence that miR-17-92 cluster deletion is linked to hepatocellular carcinoma, suggesting that these miRNAs could express tumor-suppressor properties in different contexts [43].This apparent contradiction can now be explained by considering the recently reported *ceRNA code* hypothesis, in which mRNAs combinatorially use miRNAs as a mechanism of crosstalk. Thus, competing endogenous RNAs (ceRNAs) may compete for the same miRNA, thereby affecting each other’s expression levels and being affected by the expression of the miRNA. The key point is therefore the expression level of the miRNA, suggesting that any alteration in the miRNA dose would affect the physiology of the cell leading to pathological states [44].

Speculating on the relationship between miR-19a/b expression levels and leukemogenesis, we hypothesized that lower levels of miR-19a/b could be related to cancer advancement, whereas higher expression, coupled with overexpression of BARD1 oncogenic isoforms, may provide a better response to Vorinostat treatment by altering the *ceRNA code*. In this scenario, the oncogenic role played by miR19a/b, described by Xu et al, in human cervical cancer cells could be explained [45]. The fact that Vorinostat treatment increases miR-19a/b and that cells overexpressing miR-19a/b are particularly sensitive to Vorinostat stimulation, leading to hyperactivation of caspase-9, strongly endorse this hypothesis. Moreover, considering that one of the reported miR-19b targets is Bim, a protein of the mitochondrial apoptosis pathway [42], a reorganisation in the events leading to caspase-9 activation might be postulated. The evidence that high expression levels of truncated BARD1 isoforms together with Vorinostat induction of miR-19a/b could represent a marker of response to Vorinostat in AML suggests the potential application of miR-19a/band truncated BARD isoform expression as markers in AML. In other words, it is tempting to speculate on a possible patient stratification on the basis of expression levels of miR-19 and BARD1 isoforms to better define patients that might actually benefit from epi-based treatment. Corroborating this hypothesis, BARD1 silencing in AML cells led to higher sensitivity to Vorinostat stimulation. These findings strengthen our conclusions regarding the prognostic role of miR-19a and miR-19b, and give new connotations to the isoforms of BARD1, which may prove useful both as diagnostic and predictive markers.

Moreover, the fact that *in vivo* BARD1 isoforms are expressed and significantly down- regulated in many, but not all, AML patients undergoing Vorinostat administration, strongly supports the hypothesis that patients might benefit from a pre-selection based on expression of truncated BARD1 isoforms for a more effective epigenetic treatment.

## Supporting Information

Figure S1 **Expression of BARD1 isoforms in Human blood cancer cell lines.** Cell lines: MCF7 - breast cancer; ML-2, HEL, HL-60 – blood cancer. Upper panel: BARD1 RT-PCR using forward primer from exon 1 (ATG CCG GAT AAT CGG CAG CC) and reversed primer from exon 11 (CGA ACC CTC TCT GGG TGA TA), 35 cycles. The isoforms corresponding to the amplified fragments are marked at the left. Lower panel: BARD1 RT-PCR using forward primer from exon 6 (AGC AAG TGG CTC CTT GAC AG) and reversed primer from exon 11 (CGA ACC CTC TCT GGG TGA TA), 25 cycles. GAPDH RT-PCR has been used as internal control.
(PDF)

Figure S2 **BARD1 expression can be affected by epigenetic drug treatment in human AML cell lines.** (A) BARD1 RT-PCR in human leukemia cell lines after Vorinostat treatment (5 µM). (B) BARD1 RT-PCR after 6 h Vorinostat (5 µM) treatment in 3 different human leukemia cell lines. (C) BARD1 RT-PCR in NB4 cell lines after treatment with different epi-compounds for 24 h at the indicated concentrations. GAPDH represents loading control.
(PDF)

Figure S3 **BARD1 expression is also modulated by Vorinostat treatment in solid cancer cells.** (A) BARD1 RT-PCR in HeLa, MCF7 and Kelly cells. (B) BARD1 RT-PCR in the same cell lines with a longer PCR protocol. GAPDH represents loading control.
(PDF)

Figure S4 **Validation of stable transfected clones for miR-19a and miR-19b.** Real-Time PCR for miR-19a and miR-19b in pCMV-MIR stable transfected U937 cells. Data show the mean value of three parallel experiments with error bars showing the standard deviations on top of each column.
(EPS)

Figure S5 **Visualization of BARD1ω fused to the biotin tag in HEK293T cells.** BARD1ω1 fused to N-terminal biotin tag (ω1-bioTag) was visualized with streptavidin conjugated to DyLight 488 at 20x magnification. Non-transfected cells shows weak peri-nuclear staining due to endogenously biotinylated proteins localized mostly in mitochondria. Note predominantly nuclear localization of BARD1ω1-bioTag and increased size of the nuclei expressing BARD1ω1-bioTag.
(PDF)

Figure S6 **Exogenous and endogenous expression of ω isoforms.** (A) Protein extracts from HEK293T cells transfected with pcDNA3.1 empty vector or with pcDNA3.1-Omega-A BirA tagged construct. Western blot was probed with Avidin-HRP conjugate shows a protein of expected size for Omega-A BirA and two smaller degradation products. (B) Western blot of NB4 and HL60 cells untreated (controls) and SAHA treated, show proteins of sizes corresponding to ω and degradation products that are reduced upon SAHA treatment, when probed with BARD1 C-terminal antibody (C-20). Note C-20 should recognize all BARD1 isoforms.
(PDF)
