# Supplementary figures and images for: HDAC Inhibitors Repress BARD1 Isoform Expression in Acute Myeloid Leukemia Cells via Activation of miR-19a and/or b
Source: PLoS One. 2013 Dec 11;8(12):e83018. doi: 10.1371/journal.pone.0083018 (PMC3859623; doi:10.1371/journal.pone.0083018)

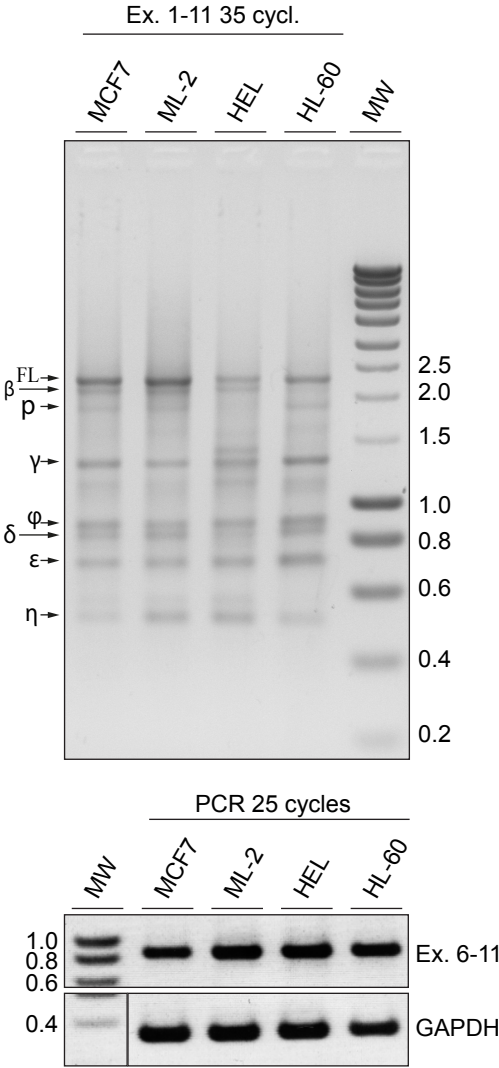

Supplement: Figure S1 — Expression of BARD1 isoforms in Human blood cancer cell lines. Cell lines: MCF7 - breast cancer; ML-2, HEL, HL-60 – blood cancer. Upper panel: BARD1 RT-PCR using forward primer from exon 1 (ATG CCG GAT AAT CGG CAG CC) and reversed primer from exon 11 (CGA ACC CTC TCT GGG TGA TA), 35 cycles. The isoforms corresponding to the amplified fragments are marked at the left. Lower panel: BARD1 RT-PCR using forward primer from exon 6 (AGC AAG TGG CTC CTT GAC AG) and reversed primer from exon 11 (CGA ACC CTC TCT GGG TGA TA), 25 cycles. GAPDH RT-PCR has been used as internal control. (PDF) [file pone.0083018.s001.pdf]

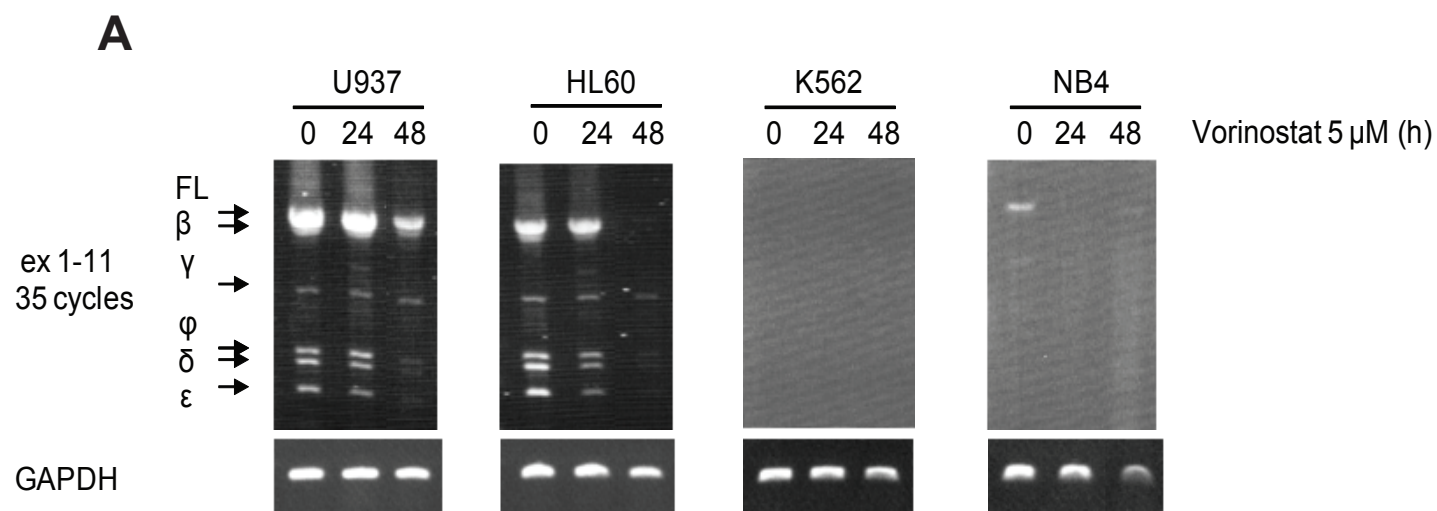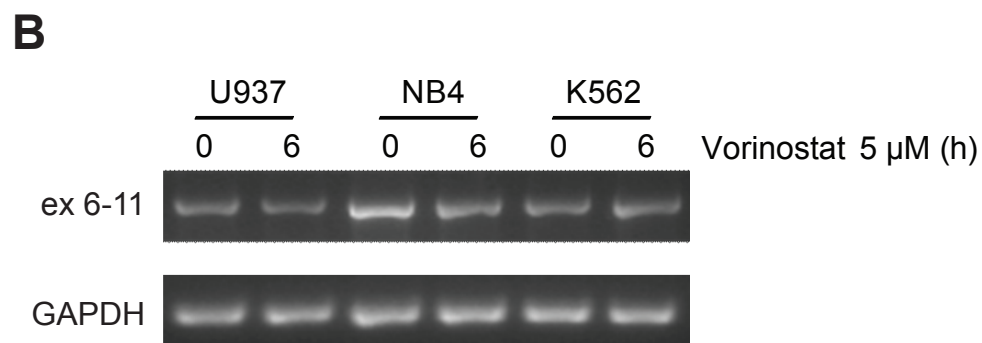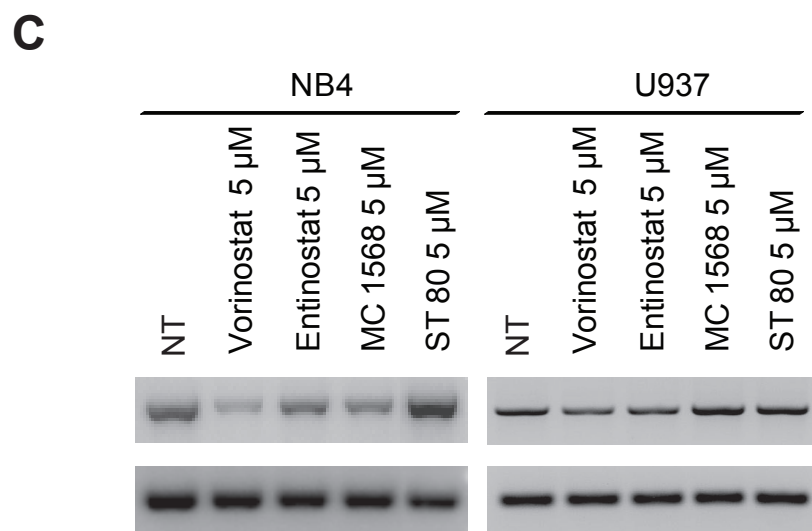

Figure S2

Supplement: Figure S2 — BARD1 expression can be affected by epigenetic drug treatment in human AML cell lines. (A) BARD1 RT-PCR in human leukemia cell lines after Vorinostat treatment (5 µM). (B) BARD1 RT-PCR after 6 h Vorinostat (5 µM) treatment in 3 different human leukemia cell lines. (C) BARD1 RT-PCR in NB4 cell lines after treatment with different epi-compounds for 24 h at the indicated concentrations. GAPDH represents loading control. (PDF) [file pone.0083018.s002.pdf]

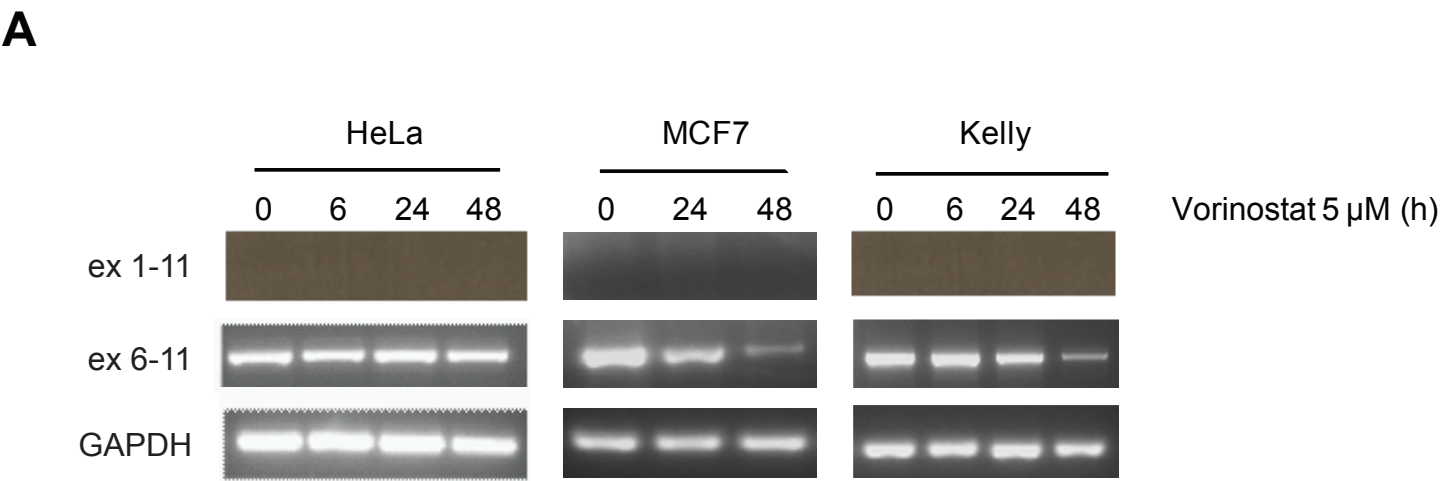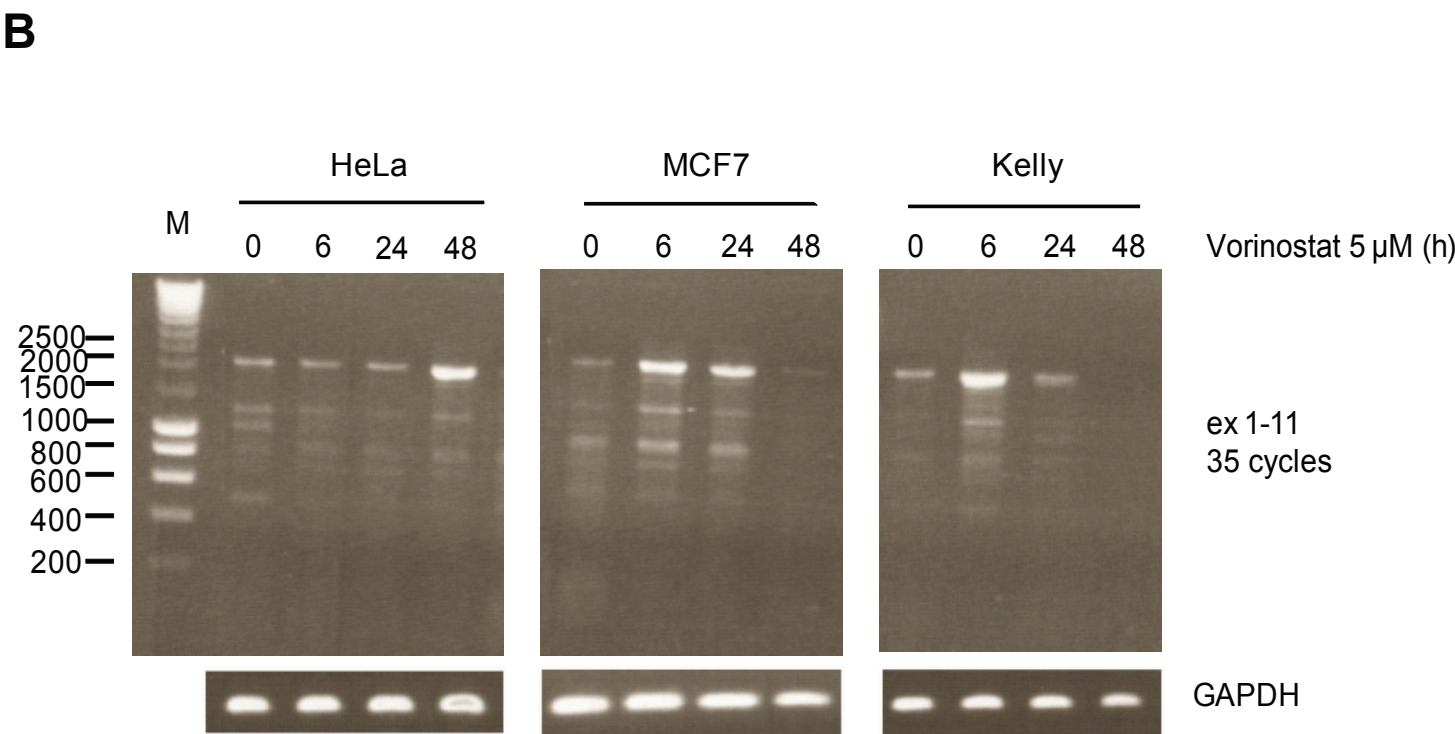

Figure S3

Supplement: Figure S3 — BARD1 expression is also modulated by Vorinostat treatment in solid cancer cells. (A) BARD1 RT-PCR in HeLa, MCF7 and Kelly cells. (B) BARD1 RT-PCR in the same cell lines with a longer PCR protocol. GAPDH represents loading control. (PDF) [file pone.0083018.s003.pdf]

DAPI

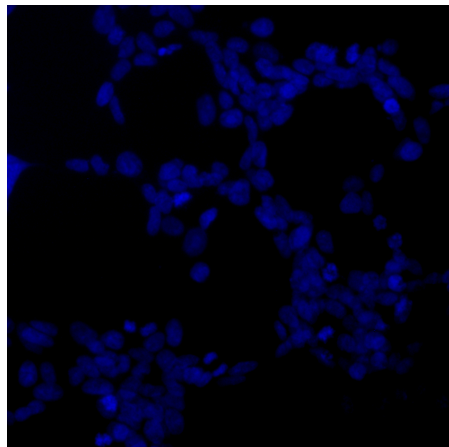

$\omega$ 1-bioTag

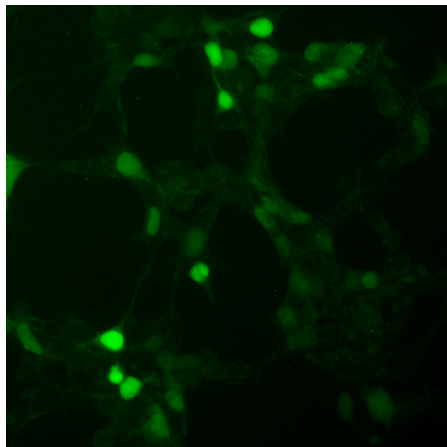

Merge

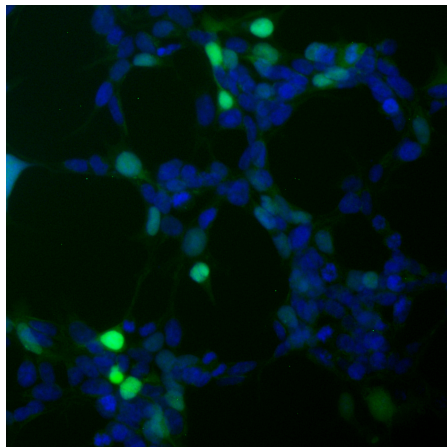

Supplement: Figure S5 — Visualization of BARD1ω fused to the biotin tag in HEK293T cells. BARD1ω1 fused to N-terminal biotin tag (ω1-bioTag) was visualized with streptavidin conjugated to DyLight 488 at 20x magnification. Non-transfected cells shows weak peri-nuclear staining due to endogenously biotinylated proteins localized mostly in mitochondria. Note predominantly nuclear localization of BARD1ω1-bioTag and increased size of the nuclei expressing BARD1ω1-bioTag. (PDF) [file pone.0083018.s005.pdf]

Figure S6

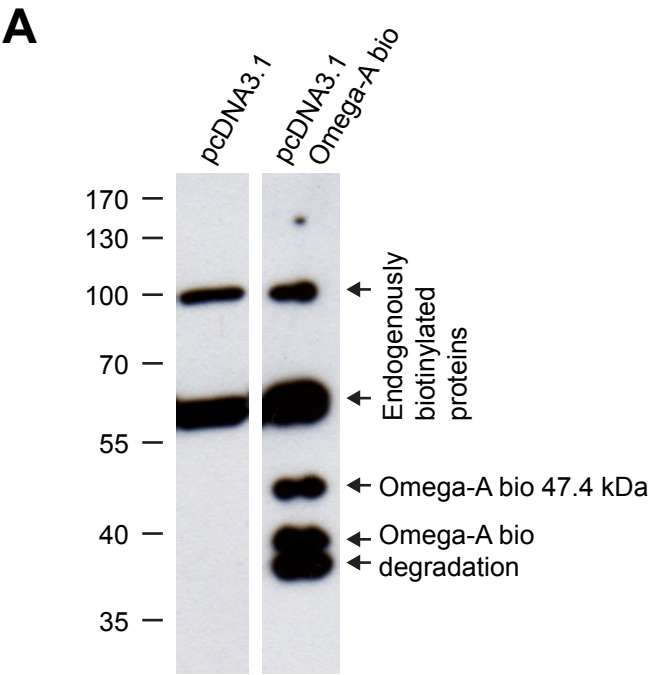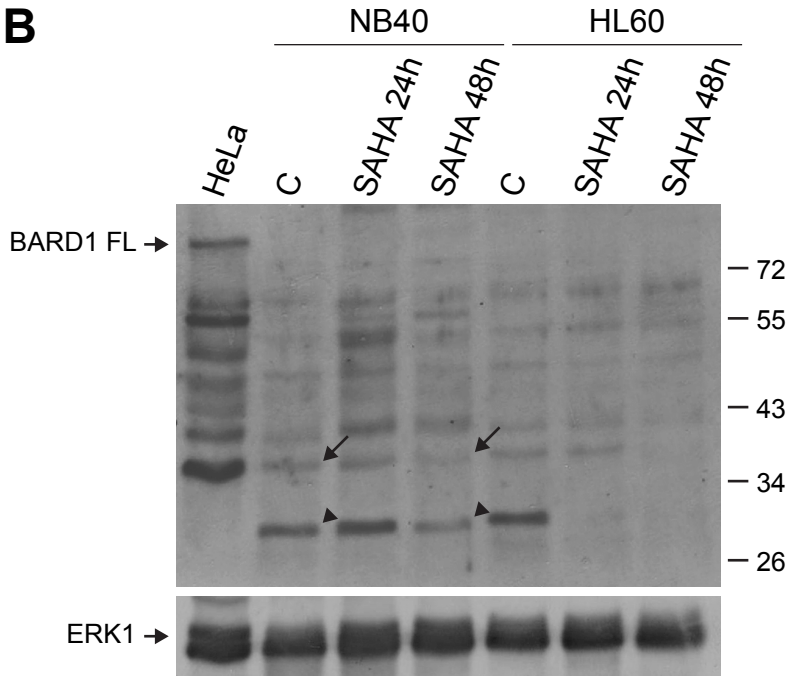

Supplement: Figure S6 — Exogenous and endogenous expression of ω isoforms. (A) Protein extracts from HEK293T cells transfected with pcDNA3.1 empty vector or with pcDNA3.1-Omega-A BirA tagged construct. Western blot was probed with Avidin-HRP conjugate shows a protein of expected size for Omega-A BirA and two smaller degradation products. (B) Western blot of NB4 and HL60 cells untreated (controls) and SAHA treated, show proteins of sizes corresponding to ω and degradation products that are reduced upon SAHA treatment, when probed with BARD1 C-terminal antibody (C-20). Note C-20 should recognize all BARD1 isoforms. (PDF) [file pone.0083018.s006.pdf]
